# Uncovering a Novel Pathogenic Mechanism of *BCS1L* in Mitochondrial Disorders: Insights from Functional Studies on the c.38A>G Variant

**DOI:** 10.3390/ijms26083670

**Published:** 2025-04-12

**Authors:** Valeria Capaci, Luisa Zupin, Martina Magistrati, Maria Teresa Bonati, Fulvio Celsi, Irene Marrone, Francesco Baldo, Blendi Ura, Beatrice Spedicati, Anna Morgan, Irene Bruno, Massimo Zeviani, Cristina Dallabona, Giorgia Girotto, Andrea Magnolato

**Affiliations:** 1 Institute for Maternal and Child Health IRCCS Burlo Garofolo, 34137 Trieste, Italy; 2 Department of Chemistry, Life Sciences and Environmental Sustainability, University of Parma, 43124 Parma, Italy; 3 Department of Life Science, University of Trieste, 34127 Trieste, Italy; 4 Department of Medicine, Surgery and Health Sciences, University of Trieste, 34129 Trieste, Italy

**Keywords:** BCS1L, mitochondrial disorder, complex III, assembly chaperone, electron transfer chain

## Abstract

The *BCS1L* gene encodes a mitochondrial chaperone which inserts the Fe_2_S_2_ iron–sulfur Rieske protein into the nascent electron transfer complex III. Variants in the *BCS1L* gene are associated with a spectrum of mitochondrial disorders, ranging from mild to severe phenotypes. Björnstad syndrome, a milder condition, is characterized by sensorineural hearing loss (SNHL) and pili torti. More severe disorders include Complex III Deficiency, which leads to neuromuscular and metabolic dysfunctions with multi-systemic issues and Growth Retardation, Aminoaciduria, Cholestasis, Iron Overload, and Lactic Acidosis syndrome (GRACILE). The severity of these conditions varies depending on the specific *BCS1L* mutation and its impact on mitochondrial function. This study describes a 27-month-old child with SNHL, proximal renal tubular acidosis, woolly hypopigmented hair, developmental delay, and metabolic alterations. Genetic analysis revealed a homozygous *BCS1L* variant (c.38A>G, p.Asn13Ser), previously reported in a patient with a more severe phenotype that, however, was not functionally characterized. In this work, functional studies in a yeast model and patient-derived fibroblasts demonstrated that the variant impairs mitochondrial respiration, complex III activity (CIII), and also alters mitochondrial morphology in affected fibroblasts. Interestingly, we unveil a new possible mechanism of pathogenicity for BCS1L mutant protein. Since the interaction between BCS1L and CIII is increased, this suggests the formation of a BCS1L-containing nonfunctional preCIII unable to load RISP protein and complete CIII assembly. These findings support the pathogenicity of the BCS1L c.38A>G variant, suggesting altered interaction between the mutant BCS1L and CIII.

## 1. Introduction

Mitochondrial disorders (MDs) are a group of genetic conditions caused by mutations in mitochondrial or nuclear DNA resulting in impairment mitochondrial oxidative phosphorylation (OXPHOS) [1,2].

The mitochondrial respiratory chain is composed of multi-heteromeric complexes (I–V) that, functioning in concert, generate an electrochemical proton gradient across the internal mitochondrial membrane (complexes I–IV), the energy of which is eventually exploited to perform the condensation of ADP + P_i_ into ATP (complex V). Due to the essential function of OXPHOS for energy production in virtually all cells, MDs often exhibit a multi-systemic involvement, heterogeneous phenotypes, and a broad spectrum of clinical presentations, although the tissues with high energy demands, such as brain and cardiac or skeletal muscles are the most affected [3].

An important subgroup amongst MDs comprises the mitochondrial complex III (CIII) deficiencies. CIII is a key component of the mitochondrial respiratory chain, since it catalyzes the transfer of electrons from coenzyme Q_10_ to cytochrome c, liberating energy exploited by the same complex III to pump protons from the mitochondrial matrix to the intermembrane space, thus sustaining the creation of the mitochondrial respiratory proton gradient. CIII consists of 11 subunits, most of which are encoded by nuclear genes, while only cytochrome b is encoded by mitochondrial DNA.

Pathogenic variants in genes encoding the core subunits of CIII and the assembly factors specific to complex III can cause mitochondrial CIII deficiency. Mutations in the *BCS1L* gene, encoding for the BCS1L protein, are the most frequent [4].

BCS1L is a homo-heptameric transmembrane AAA-ATPase belonging to the superfamily of AAA proteins. It acts as a chaperone, allowing the translocation of the folded Fe_2_S_2_ iron–sulfur Rieske (RISP) protein across the inner mitochondrial membrane, and its incorporation in the CIII precomplex [5,6,7,8].

The *BCS1L* phenotypic spectrum ranges from Björnstad syndrome (BJS, OMIM #262000), characterized by noncongenital sensorineural hearing loss (SNHL) and *pili torti*, to GRACILE syndrome (Growth Retardation, Aminoaciduria, Cholestasis, Iron Overload, and Lactic Acidosis, OMIM #603358) at the most severe end of the spectrum, through several different clinical manifestations in between, including proximal renal tubular acidosis (RTA), SNHL, and developmental delay [9,10].

Genetic mutations in *BCS1L* may affect BCS1L protein levels and its mitochondrial import, the assembly/stability of CIII and supercomplexes, as well as the mitochondrial network [5,6]. However, the molecular mechanisms underlying the pathogenicity of specific variants remain incompletely understood.

This study investigates the pathogenicity of a *BCS1L* variant found in a child with a phenotype suggestive of an MD through functional assays in both yeast models and patient-derived human dermal fibroblasts (HDFs).

## 2. Results

### 2.1. Clinical Report

The patient, 27 months at the last follow-up, was the first-born offspring of healthy unrelated Italian parents of Caucasian ethnicity coming from a small city in northern Italy. He was born at term by vaginal delivery after spontaneous normal pregnancy. Family history was unremarkable. Apgar scores were 9 and 10 at 1 and 5 min. His birth weight was 2680 g (−2.04 SDS, small for gestational age, SGA), birth length 48 cm (−1.61 SDS), and occipitofrontal circumference 33 cm (−1.56 SDS). At 35 weeks of gestation, a prenatal ultrasound detected an anomaly in the renal regions. Following birth, a renal ultrasound confirmed bilateral renal ectopy, with both kidneys located in the right pelvic cavity. A subsequent evaluation of renal function revealed normal results, suggesting that this might represent a finding unrelated to his pathology.

He failed the newborn hearing screening, and as recommended by guidelines worldwide, a CMV diagnosis was ruled out using urine PCR analysis. At the age of 2 months, bilateral moderate-severe neurosensorial hearing loss was diagnosed as a result of otoemissions absence and pathological acoustic evoked potentials (V wave identifiable up to a stimulation threshold of 60 dB nHL bilaterally). Because of sensorineural hearing loss, trio whole exome sequencing (WES) was performed.

Following a reverse phenotyping approach, at the age of 18 months, the proband was found to be affected by proximal renal tubular acidosis, liver damage with high levels of plasma transaminases (4-fold normal values), and failure to thrive (weight 10.5 Kg, −0.37 SDS; length 71 cm, −4.17 SDS; BMI 20.83 Kg/m^2^, 3.02 SDS). Brain MRI and internal auditory structures were normal, whereas slight opacities of the lens were identified at eye examination. Echocardiogram was normal. He exhibited woolly and hypopigmented/brittle hair; however, *pili torti* were excluded at hair analysis by optical microscope.

At 23 months of age, the proband’s growth parameters were weight 10.7 Kg (−1.02 SDS), height 77.5 cm (−3.24 SDS), and BMI 17.81 Kg/m^2^ (0.79 SDS). A stimulation test with arginine was performed to assess growth hormone levels, which were found to be within the normal range (peak of 13.6 ng/mL). To correct the metabolic acidosis, oral supplementation with bicarbonate was started. However, despite a progressive incrementation of oral bicarbonates from 5 mEq/kg/die to 15 mEq/kg/die, blood tests showed persistent low levels of serum bicarbonates and severe acidosis. Management of the therapy was also complex due to poor patient compliance and a percutaneous endoscopic gastrostomy (PEG) was placed obtaining acid base balance by enteral administration of bicarbonates and other electrolytes.

He exhibited a delay in psychomotor development: he sat at 5 months, stood alone at around 18 months, and walked unassisted at 25 months. At the age of 18 months, his development was measured by Bayley III to be age-appropriate for the cognitive area (Development Quotient, DQ, 95) with weakness in the language skills (DQ 59), both receptive and expressive; however, the motor area was below the norm with deficit in gross motor, and static and dynamic balance skills, DQ 70. He was able to say his first words at 23 months and to combine two words at 27 months, exhibiting a sudden ‘lexical explosion’. At a speech and language assessment at 27 months, supported by administering standardized questionnaires (i.e., Little Ears, IT_MAIS and MacArthur-Bates CDI), language skills were measured to be slightly delayed. However, the patient had useful non-verbal communication to express needs and mood/emotions. Scores of the Newcastle Paediatric Mitochondrial Disease Scale (NPMDS) 0–24 months are shown in Supplementary Table S1. Items contributing the scores refer to developmental delay, in which gross motor skills were more impaired than language delay, fitting with the Bayley III results, and renal disease.

### 2.2. Genetic Evaluation

**Figure 1:**
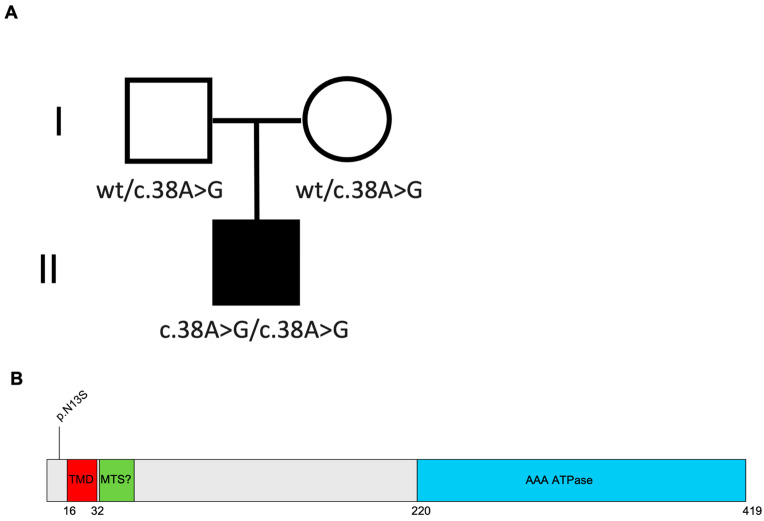
Case report. (**A**) Pedigree of the family. The black symbol indicates the subject carrying the homozygous variant c.38A>G in BCS1L (NM_004328.5) gene. The parents are carriers of the same variant. (**B**) Schematic structure of BCS1L protein NP_001073335.1 indicating the aminoacidic substitution.

The WES analysis showed homozygous for the *BCS1L* variant (NM_004328.5): c.38A>G, p.(Asn13Ser). Both parents were carriers of the same variant (Figure 1A).

The p.(Asn13Ser) missense variant was of uncertain significance (VUS) according to the ACGS/ACMG criteria PM1 and PM2_moderate, PS3_supporting [11] (https://wintervar.wglab.org, accessed on 2 January 2024). The variant was absent from the gnomAD population database (https://gnomad.broadinstitute.org, accessed on 2 January 2024).

It was previously described in a male affected by ventilation insufficiency, SNHL, tubulopathy, and hepatopathy; he died at 11 months (personal communication from Prof. Saskia B. Wortmann) [10]. Indeed, the *BCS1L* variant (NM_004328.5): c.38A>G, p.(Asn13Ser) was reported as pathogenic in Varsome (https://varsome.com, accessed on 2 January 2024) and in HGMD^®^ Professional (https://digitalinsights.qiagen.com, accessed on 2 January 2024) (Figure 1B).

The Asn13 amino acid position is highly conserved from human to *Saccharomyces cerevisiae*. It is located in the putative mitochondrial targeting signal, which is not cleaved in the case of *BCS1L* [12,13].

### 2.3. Yeast Complementation Assay Confirmed BCS1L c.38A>G Variant Pathogenicity

To study the functional effect of the p.Asn13Ser variant identified in the patient, we exploited the yeast *S. cerevisiae* ortholog gene *BCS1*.

As it was previously demonstrated that human *BCS1L* cDNA is able to complement the defect of the yeast *bcs1Δ* null mutant [6,14,15], we used a homologous and a heterologous complementation approach, the latter consisting of the expression of the human *BCS1L* gene (wild-type or harboring the Asn13Ser variant), cloned into the pYEX expression vector, in the yeast *bcs1Δ* strain.

**Figure 2:**
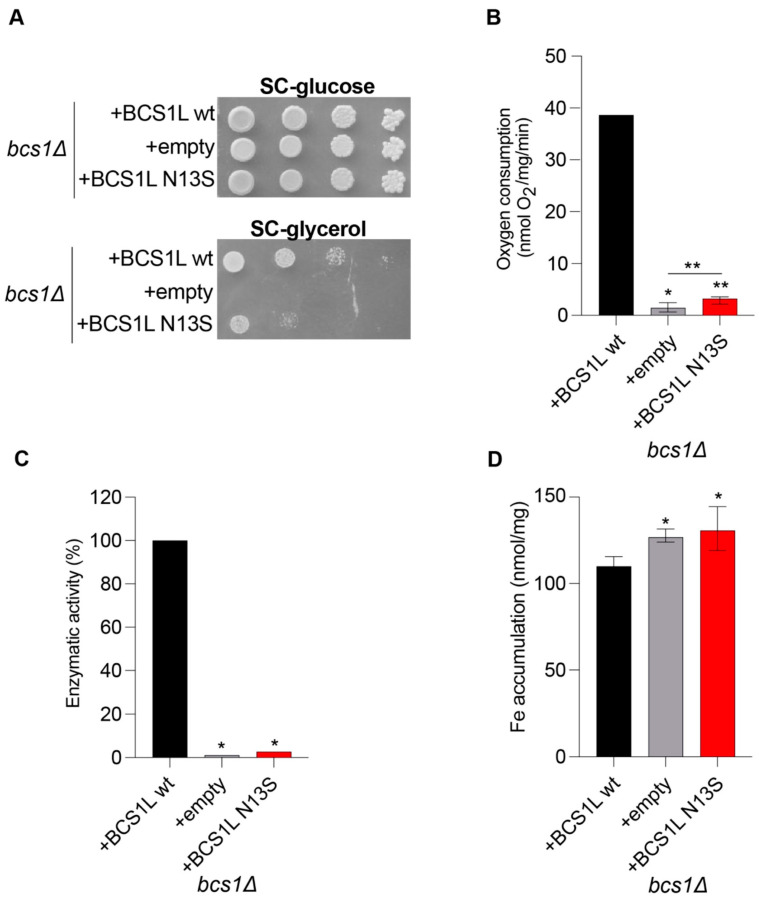
Heterologous complementation study in Saccharomyces cerevisiae using the human BCS1L cDNA. (**A**) Growth test: a bcs1Δ yeast strain harboring either the wild-type human BCS1L, the mutant allele (bcs1lN13S), or the empty vector were serially diluted and spotted on SC agar plates supplemented with the fermentable carbon source glucose (2%) or the non-fermentable carbon source glycerol (2%) and incubated at 36° C. (**B**) Respiratory activity: yeast strains were grown at 36 °C in SC medium supplemented with 0.6% glucose. Data are the mean of at least three values ± SD. The black bar indicates the wild-type strain; the grey bar indicates the strain carrying the alleged pathological mutation; the white bar indicates the null mutant strain. Statistical analysis was performed using the Mann–Whitney test: * *p* < 0.05; ** *p* < 0.01. (**C**) NADH-cytochrome c oxidoreductase (NCCR) activity: recorded on a mitochondrial-enriched fraction from yeast strains grown at 36° C in SC medium supplemented with 0.6% glucose. Data were normalized to the wild-type and represented as the mean of at least four values ± SD. Statistical analysis was performed using the Mann–Whitney test: * *p* < 0.05. (**D**) Iron accumulation: quantified in yeast strains grown up to the early stationary phase in SC medium supplemented with 0.6% glucose; 2 mM of ferrous sulfate was added. Statistical analysis was performed using the Mann–Whitney test: * *p* < 0.05.

Data obtained by spot assay analyses and oxygen consumption measurements show a strong, albeit partial, oxidative growth defect and respiratory activity reduction for the strain carrying the mutant *bcs1l^N13S^* allele (Figure 2A,B). To better characterize the mechanisms underlying the mitochondrial impairment, we also showed that the *bcs1l^N13S^* variant determines a strong CIII activity defect (Figure 2C).

To evaluate if the mutation Asn13Ser is associated with iron accumulation, a quantitative determination of iron levels after growth with 2 mM ferrous sulfate was performed by a colorimetric assay that relies on the formation of colored iron complexes with BPS after nitric acid digestion of yeast cells [16]. A significant increase in iron levels was observed in the strain carrying bcs1l^N13S^ (Figure 2D).

**Figure 3:**
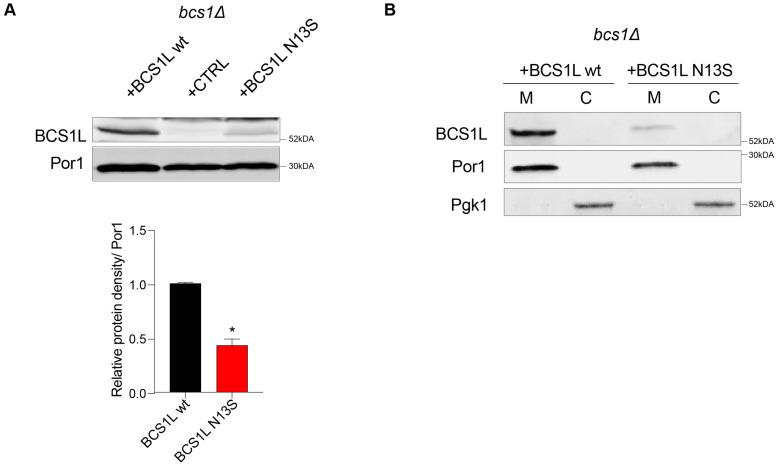
Protein analyses in *Saccharomyces cerevisiae.* (**A**) Protein quantification: Representative Western blot on total protein extract using antibodies recognizing Bcs1l or Por1 as loading control; signals were first normalized to Por1 and then to the wild-type signal to which the value 1.0 was assigned. Densitometric analysis is reported in the histogram below the blot and was performed on at least three independent blots using Image Lab Software v6.1 (Bio-Rad, Hercules, CA, USA). Statistical analysis was performed using the Mann–Whitney test: * *p* < 0.05. (**B**) Proteins localization: Western blot on denaturing SDS–PAGE of mitochondrial (M) and cytosolic (C) proteins from *bcs1Δ* strains expressing wild-type *BCS1L* or *bcs1l^N13S^*. An antibody against Bcs1l was used to detect Bcs1l; antibodies against Por1 and Pgk1 were used as markers of the mitochondrial and cytosolic fractions, respectively.

Furthermore, we assessed the effect of the variant on protein quantity, by measuring the steady-state level of the bcs1l wild-type and mutant protein through Western blot analysis. A significant reduction in the protein quantity was observed in the strain carrying *bcs1l^N13S^* (43% ± 6 relative to the wild type), suggesting that the variant severely alters protein stability (Figure 3A).

Due to the mutation position in the putative mitochondrial target sequence (MTS) [7], we tested whether the aminoacidic change could inhibit the correct protein localization into mitochondria. Separation of cytosolic and mitochondrial proteins followed by Western blot analyses was employed to verify this hypothesis. The antibodies against porin/VDAC1 (Por1) and phosphoglycerate kinase (Pgk1) were used as markers for the mitochondrial and cytosolic fractions, respectively. Both the wild-type bcs1l and mutant bcs1l*^N13S^* proteins correctly localized into the mitochondria as demonstrated by the signal in the mitochondrial fraction (Figure 3B). Due to differences in the mitochondrial targeting sequence between yeast and humans and therefore possibly in the mechanism of protein import, we also analyzed protein localization of the yeast Bcs1^N49S^ mutant protein. As no commercial antibody against yeast Bcs1 exists, the HA epitope was added by mutagenic overlap PCR at the 3′ end. This data indicate that the mutation does not interfere with protein import but rather affects protein function and/or quantity.

In addition, a homologous complementation approach, using the yeast *BCS1L* ortholog gene *BCS1*, was used. As shown by protein alignment (Supplementary Figure S1A), the human residue Asn13 is conserved and corresponds to the yeast residue Asn49. Thus, the corresponding codon was mutagenized, producing the mutant allele *bcs1^N49S^*. The *BCS1* wild-type allele or the mutant allele, cloned into the centromeric vector pFL38, were transformed into the yeast *bcs1Δ* null mutant, thus obtaining the strains *bcs1Δ*/*bcs1* and *bcs1Δ*/*bcs1^N49S^*, respectively. The *bcs1Δ* strain was also transformed with the empty vector as a control, obtaining *bcs1Δ*/*pFL38*. The latter strain could not grow on non-fermentable carbon sources (e.g., glycerol); however, re-expression of the wild-type *BCS1* rescued the growth defect (Supplementary Figure S1B). The expression of the *bcs1^N49S^* mutant allele resulted in a severe oxidative growth defect, but a partial ability to grow on oxidative carbon sources was retained (Supplementary Figure S1B).

To better characterize the oxidative defect, mitochondrial respiratory activity was assessed by measuring the oxygen consumption rate; the strain expressing the wild-type *BCS1* allele consumed 42.55 ± 7.33 nmol O_2_/mg/min. Instead, the strain expressing the mutant *bcs1^N49S^* allele showed a strong reduction of oxygen consumption (4.87 ± 1.08 nmol O_2_/mg/min), but a partial respiratory activity was retained. In fact, a significant difference with the null mutant strain (2.38 ± 0.67 nmol O_2_/mg/min) was recorded (Supplementary Figure S1C). Together, the results obtained show that the yeast Asn49Ser variant, equivalent to the human mutation Asn13Ser, is deleterious in yeast, supporting its pathogenicity in humans.

Altogether, data obtained in the yeast of the BCS1L Asn13Ser variant affecting oxidative growth and mitochondrial respiratory activity demonstrate its pathogenicity.

### 2.4. BCS1L N13S Affects Mitochondrial Respiration and CIII Activity in Human Dermal Fibroblasts (HDF)

The variant’s pathogenicity shown in the yeast model prompted us to extend the analysis in human cell models.

**Figure 4:**
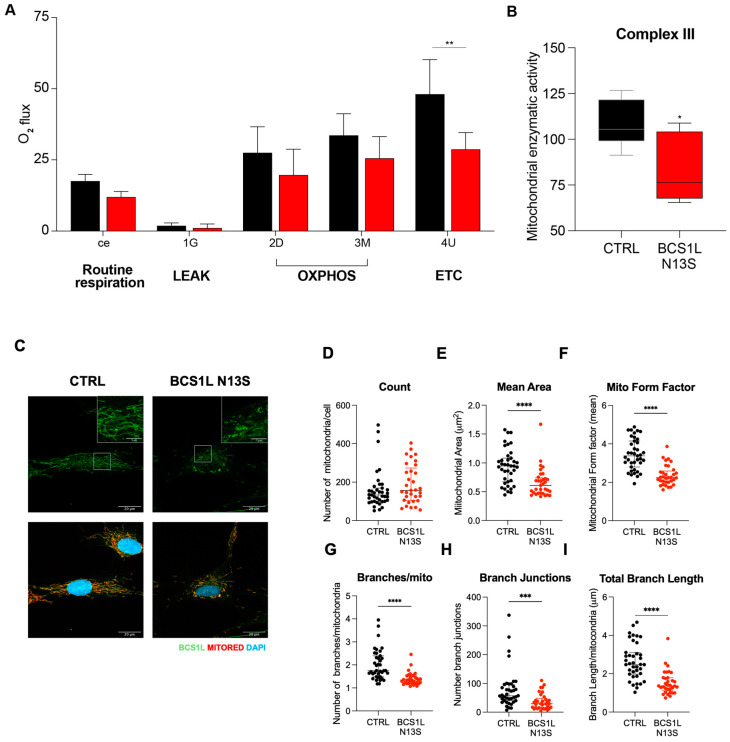
BCS1L N13S affects mitochondrial metabolic activity and morphology in human dermal fibroblasts (HDFs). (**A**) Specific O_2_ flux and (**B**) enzymatic activity of CIII normalized to the activity of citrate synthase in HDF primary cells, in either the control or mutated BCS1L N13S. Graph represents the mean ± SEM of N ≥ 3 independent experiments. (**C**) Representative images of immunofluorescence analysis of BCS1L (green) in in HDF primary cells in either the control or mutated BCS1L N13S. Mitochondria were counterstained with mitochondrial marker MITORED (red) and nuclei DAPI staining (blue) Scale bar 20 mm (inset scale bar 5 mm). (**D**–**I**) Graphs show mitochondrial count, area, forma factor and branches/mitochondria, branches junction, and length calculated by Mitochondrial Analyzer image J Plugin. (*n* = 35 cells for each condition). *p* value (* *p* < 0.05, ** *p* < 0.01, *** *p* < 0.001, **** *p* < 0.0001) was calculated by paired two-tailed Mann–Whitney test.

We isolated primary human dermal fibroblasts (HDFs) from the patient’s skin punch biopsy. In HDF cells, we first performed high-resolution respirometry analysis by Oroboros O2k oxygraphy showing a reduction of oxygen consumption in BCS1L Asn13Ser HDFs cells as compared to control wild-type cells (Figure 4A). In detail, “routine respiration” was similar between the controls and the patient. Similarly, the non-phosphorylating resting state (“LEAK respiration”) stimulated by the addition of glutamate, and the OXPHOS capacity investigated through the oxidation of NADH at the level of CI stimulated by the addition of ADP and malate, did not display significant changes between control and patient-derived HDF cells, even though a slight reduction was observed. However, the electron transfer capacity (noncoupled electron transfer-state) activated by CCCP was lower in the patient’s cells as compared to a control, indicating an electron chain transport impairment. Similar results on oxygen consumption were obtained upon replacement of glucose with galactose in the culture media to force cells to rely upon OXPHOS for ATP production (Supplementary Figure S2C); indeed, BCS1L Asn13Ser HDF cells grown in galactose show a strong reduction of proliferation as compared to the control, as well as to cells grown in glucose-rich medium (Supplementary Figure S2D).

Taken together, the results clearly indicate impairment in electron transport. We then analyzed the activities of the mitochondrial respiratory chain enzymes, NADH dehydrogenase (complex I), DBH2:cytochrome oxidoreductase reductase (complex III), and cytochrome c oxidase (complex IV). Spectrophotometric kinetic measurement showed a strong reduction of the CIII enzymatic activity (Figure 4B, *p* = 0.04) (Supplementary Figure S2A).

Altogether, these data demonstrate that the BCS1L Asn13Ser variant causes a reduction of oxygen consumption and a severe decrease of complex III activity in patient-derived fibroblasts confirming the deleterious impact of the variant as suggested by the yeast model.

### 2.5. BCS1L N13S Alters Mitochondrial Morphology and Network in HDFs

In addition, previous reports showed that some BCS1L protein variants might induce cytoplasmic protein mislocalization as well as abnormal mitochondrial morphologies secondary to the OXPHOS deficiency [4,15].

To verify the impact of the Asn13Ser variant, we performed immunofluorescence of HDFs derived either from the patient or control, finding that both BCS1L, either wild-type or Asn13Ser, specifically localized at the mitochondria, as indicated by Mitotracker Red mitochondrial marker co-staining (Supplementary Figure S2B).

In addition, in patient fibroblasts, we assessed mitochondrial morphology by Mitotracker staining and BCS1L immunofluorescence, showing punctate mitochondria, shorter tubules, and reduced branches (Figure 4C). Interestingly, we did not find any significant differences in mitochondrial count (Figure 4D), while the average area and form factor (that reflects the complexity and branching aspect of mitochondria) were reduced in patient cells (Figure 4E,F), suggesting smaller and more fragmented mitochondria. In addition, analyses of the mitochondrial network showed a reduction in the number, junction, and length of mitochondrial branches in BCS1L Asn13Ser HDF cells (Figure 4G–I).

Altogether, these data suggest an impairment of the mitochondrial network in patient’s fibroblasts harboring the BCS1L Asn13Ser variant.

### 2.6. BCS1L N13S Affects CIII Assembly

Importantly, data obtained in yeast suggest that the BCS1L Asn13Ser variant affects protein levels. To validate this effect in human cells, we performed an immunoblot of BCS1L in lysates obtained from control- or patient-derived HDF cells, finding a significantly strong reduction of BCS1L levels (around 50%, *p* = 0.004), without altering the levels of RISP protein, while cytochrome-b was slightly reduced (25%, *p* = 0.04) (Figure 4A), suggesting that the whole complex III holoenzyme is reduced. Of note, differences found in BCS1L protein levels does not rely on *BCS1L* gene expression changes since mRNA levels in HDFs are unaltered (Supplementary Figure S3B).

To understand the molecular mechanism underlying the BCS1L Asn13Ser-dependent biochemical alterations, we evaluated its impact on complex CIII assembly.

**Figure 5:**
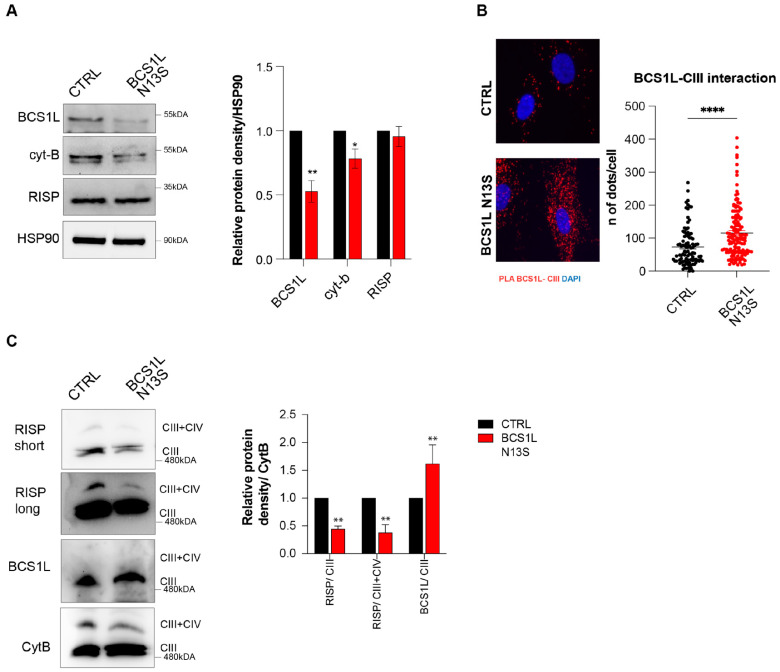
BCS1L N13S affects CIII assembly. (**A**) Representative images of Western blot analysis of the BCS1L in HDF primary cells in the either control or mutated BCS1L N13S. Graph shows the means ± SEM of three independent experiments, where the relative expression level of the proteins was obtained by densitometry measures and normalized to HSP90. (**B**) Representative images of PLA between BCS1L and CIII in HDF cells. Right: Graph showing the number of dots/cell. (*n* ≥ 100 cells/condition). (**C**) Representative images of BN-PAGE of digitized HDF primary cells in either the control or mutated BCS1L N13S followed by Western blot analysis of RISP, BCS1L, and Cyt-b proteins. Right: Graph shows the means ± SEM of three independent experiments, where the relative expression level of the proteins was obtained by densitometry measures and normalized to Cyt-B. *p* value (* *p* < 0.05, ** *p* < 0.01, **** *p* <0.0001) was calculated by an unpaired two-tailed Mann–Whitney test.

As a first step, we assessed the interaction between BCS1L and CIII by proximity ligation assays; unlike what was expected, the binding of BCS1 to the CIII complex was increased with the BCS1 Asn13Ser mutant form in HDFs as compared to the control (Figure 5B).

To avoid genetic background effects, plasmids overexpressing FLAG-tagged BCS1L, either wild-type or a N13S mutant, were transfected into HDF cells from patients and controls and a proximity ligation assay was performed. The results showed that, regardless of the genetic background, BCS1L N13S interacts more with CIII (as revealed by anti- FLAG and cyt-b antibodies) compared to wild-type BCS1L, in both control and patient-derived fibroblasts (Supplementary Figure S3B).

Thus, we next characterize the CIII assembly in patient cells by WB analysis on blue native gel electrophoresis. Western blot analysis with the anti-Cyt-B antibody showed a marked reduction of the CIII-CIV complex band in patient-derived cell lysates relative to the control; accordingly, the RISP protein is less present in CIII and CIII-CIV complexes. Taken together, these results indicate a reduction of the complete assembled CIII complex. Surprisingly, mutant BCS1L protein showed an accumulation in CIII complex in fibroblasts from the patient (Figure 5C).

Altogether, these results suggest that BCS1L is unable to properly transfer the RISP protein on the nascent CIII complex remaining attached to it, resulting in a reduction of CIII holoenzyme and CIII-CIV supercomplex assembling, and the RISP protein (unaltered in total levels) is only partly bound to BCS1L remaining unincorporated.

## 3. Discussion

BCS1L is a homo-heptameric transmembrane AAA-ATPase that acts as a chaperone for CIII holoenzyme. Mutations in the *BCS1L* gene might affect BCS1L protein levels and its mitochondrial import, assembly/stability of CIII and supercomplexes, and the mitochondrial network [5,6].

In this work, we identified a homozygous c.38A>G variant in the BCS1L gene (p.Asn13Ser) in a 27-month-old male showing proximal renal tubular acidosis, sensorineural hearing loss, hypopigmented hair, and developmental delay [4]. By this clinical picture, the patient did not fulfill clinical criteria to perform classical GRACILE or Björnstad syndrome diagnosis. Although exhibiting clear symptoms of mitochondriopathy, its specific disease requires reclassification. Indeed, BCS1L-related diseases have a wide range of clinical symptoms that manifests as a continuum spectrum, instead of being distinct clinical entities [10], and is referred to as mitochondrial complex III deficiency, nuclear type 1 (MC3DN1, OMIM #124000).

The p.Asn13Ser missense mutation localizes in the N-terminus of the BCS1L protein. The N-ter of BCS1L consists of three specialized regions involved in targeting and sorting of the protein in the mitochondria, namely the transmembrane domain (TMD), the mitochondrial targeting sequence (MTS), and the import auxiliary sequence (IAS) [12,13]. The residue 13 localizes in a portion of the protein that does not form any structural domain and lies in the intermembrane space. Thus, in analogy to other reported variants in the N-ter of *BCS1L*, the mutation might affect BCS1L proper mitochondrial localization; however, in HDF cells, by BCS1L immunostaining and mitochondrial counterstaining, we showed that the BCS1L Asn13Ser does not influence it, excluding this as the pathogenic mechanism induced by the variant. Conversely, the mutant form of BCS1L seems to be less stable than the wild-type counterpart since the levels of BCS1L protein are reduced while its mRNA levels are unchanged. These results were supported by data in yeast models, that recapitulate a reduction on protein levels without affecting the mitochondrial localization; these results support an essential, evolutionary conserved role of the pAsn13 residue in the stability of the BCS1L protein.

Functional analysis, both in yeast models and in patient-derived human dermal fibroblasts, further unveiled the pathogenicity of this BCS1L variant showing the impairment of the mitochondrial respiratory function and the decrement of CIII activity, accompanied also by disruption of the mitochondrial network.

BCS1L is an assembly factor required for the insertion of the Rieske Fe_2_S_2_ protein into the CIII precomplex to complete CIII assembly. Thus, defects in BCS1L indeed might cause iron overload as the yeast model confirmed for the reported variant [17].

Importantly, in patient-derived cells, we found that the levels of RISP were unaltered, despite the reduction of cytochrome-b levels and CIII activity, suggesting a deleterious impact on CIII complex assembly and stability.

To shed light on this molecular mechanism, we evaluated the interaction between the BCS1L and CIII complex by proximity ligation assay (PLA). PLA is based on immunodetection of two different proteins close to each other at a distance of 20 nanometers or less. This experiment shows a marked increase of interaction between CIII and mutant BCS1L, compared to the wild-type protein, suggesting that the binding between CIII and BCS1L, which is normally transient in controls, remains stalled in the Asn13Ser BCS1L variant. WB on native gel electrophoresis confirmed an impairment of CIII holoenzyme assembly and in the formation of the CIII-CIV supercomplex. The mutant BCS1L was unable to load RISP into the nascent complex III to complete its assembly, thus resulting in the accumulation of a BCS1L-containing nonfunctional preCIII. These findings give new insights into the pathogenic mechanisms of BCS1L mutant protein.

Interestingly, the same variant was previously described in a patient with worse clinical manifestation who died at 11 months old, suggesting the influence of the genetic background and the existence of compensatory mechanisms [10].

Besides the role on CIII activity, the exact mechanism underlying the *BCS1L*-related phenotype remains unclear.

Even though the role of BCS1L as a chaperone on CIII is solid, its impact in OXPHOS in different tissue is less specific, as *BCS1L* pathogenetic variants were identified in patients exhibiting normal respiratory chain function in the skeletal muscle, skin fibroblasts, and liver [10]. This evidence suggests that other downstream cellular mechanisms might contribute to the pathomechanism and disease severity. *BCS1L* pathogenic variants affecting the assembly or stability of respiratory complexes CIII and the supercomplex assembly between CI, CIII, and CIV might lead to ROS production, cell death, and structural alterations in the mitochondrial network. Upon mitochondrial dysfunction, cells are able to trigger compensatory responses, relying on retrograde signals from the mitochondria to the nucleus [18]. Only recently, it has been reported that the BCS1L mutant causes DNA damage, cellular senescence, and systemic progeroid phenotype by triggering c-MYC upregulation, starting to shed light of the role of BCS1L in these processes [19]. Identification of such pathways might ameliorate the clinical management of the secondary symptoms caused by *BCS1L* pathogenic variants; that, however, goes further beyond the scope of this study.

Indeed, the mechanism by which this set of phenotypes is acquired is not known. More importantly, except for the Finnish variant (c.232A>G p.Ser78Gly) which always causes GRACILE syndrome with a well-defined natural history and outcome [20,21], for the other specific variants the pathogenicity remains incompletely understood without any clear correlation between the location of the mutations in the gene and the severity and clinical presentation of *BCS1L*-related disease.

In this study, we report on the biochemical and metabolic consequences of a previously uncharacterized *BCS1L* variant, resulting in mitochondrial morphology and bioenergetic impairment. Unexpectedly, we reported the increased interaction between BCS1L and CIII, indicating the accumulation of BCS1L-containing nonfunctional preCIII unable to load RISP protein and complete CIII assembly. These data characterized the mechanism of pathogenicity and expand the phenotypic spectrum of *BCS1L* c.38A>G variant.

Understanding these pathogenic mechanisms contributes to the broader knowledge of mitochondrial disorders, possibly allowing for targeted interventions, finally improving the clinical outcomes of mitochondrial patients with CIII deficiency, which remains untreatable to date. Ultimately, these efforts could expand awareness of OXPHOS defects, deepen our understanding of their complex phenotypes, and pave the way for better treatment approaches in the future.

## 4. Materials and Methods

### 4.1. BCS1L Patient

#### 4.1.1. Subject

Between 6 and 7 months of age, the proband underwent an evaluation as a second opinion at the Department of Audiology and Otorhinolaryngology, Institute for Maternal and Child Health IRCCS ‘Burlo Garofolo’, Trieste. He was then referred to a genetic consultation for bilateral moderate-severe hearing loss. Genetic tests were performed at the Medical Genetics Laboratory, Institute for Maternal and Child Health IRCCS ‘Burlo Garofolo’, Trieste (Italy), as part of routine genetic tests. Written informed consent for the genetic tests and research were obtained from the parents of the patient.

#### 4.1.2. Genetic Analysis

Genomic DNA was extracted from venous peripheral blood lymphocytes using the QIAsymphony^®^ SP instrument with the QIAsymphony^®^ DNA Midi (Qiagen, Hilden, Germany) according to the manufacturer’s instructions. WES was performed on an Illumina NextSeq 550 instrument (Illumina Inc., SanDiego, CA, USA) by applying the Twist Exome 2.0 plus Comprehensive Exome Spike-in kit (Twist Bioscience, South San Francisco, CA, USA, Variant interpreter software https://www.engenome.com (accessed on 2 January 2024) was used for variants annotation and filtering, including the CopyNumber Variations (CNVs). A final VCF file was generated for each individual and was analyzed through the enGenome Expert Variant Interpreter (eVai) software (https://evai.engenome.com, accessed on 2 January 2024) Variants were filtered as follows: (1) variants previously reported as polymorphisms both in NCBI dbSNP build 155 (https://www.ncbi.nlm.nih.gov/snp, accessed on 2 January 2024) and gnomAD (https://gnomad.broadinstitute.org, accessed on 2 January 2024) were excluded; in particular a minor allele frequency (MAF) cutoff of 0.001 was used, (2) the pathogenicity of all identified variants was evaluated through several in silico prediction tools, including PolyPhen-2 [22]. Sorting Intolerant From Tolerant (SIFT) [23], Pseudo Amino Acid Protein Intolerance Variant Predictor (PaPI score) [24], Deep Neural Network Variant Predictor (DANN score) [25], and dbscSNV score [26], (3) SNVs leading to synonymous amino acid substitutions not predicted as damaging, nor affecting splicing, or highly conserved residues were excluded, 5) variants with a quality score (QUAL) < 20 or called in off-target regions were excluded as well. Consultation of the ClinVar (https://www.ncbi.nlm.nih.gov/clinvar, accessed on 2 January 2024), the Human Gene Mutation Database professional (Qiagen), Online Mendelian Inheritance in Man (OMIM) (https://www.omim.org, accessed on 2 January 2024), and DECIPHER (https://www.deciphergenomics.org, accessed on 2 January 2024) contributed to the interpretation of selected variants. ACMG criteria have been considered for variant classification [11].

### 4.2. Yeast Model

#### 4.2.1. Yeast Strain and Growth Conditions

Yeast strain used in this work was *ade2–1 leu2 3112 ura3–1 his3–1 trp1–1 bcs1::HIS3* (*bcs1Δ*) [17].

The *BCS1* yeast gene was amplified and cloned under its natural promoter into the centromeric vector pFL38. *BCS1* was mutagenized by the PCR overlap technique to obtain the mutant allele (*bcs1*^N49S^) and cloned in the pFL38 vector. To add HA epitope at the 3′ of the wild-type *BCS1* and *bcs1*^N49S^, a PCR overlap was performed with appropriate primers and the amplicon was cloned in the pFL38 vector.

The *bcs1l^N13S^* mutant allele was generated with the PCR QuikChange technique using *BCS1L* cDNA cloned in the pYEX plasmid as a template [6].

All the obtained plasmids and the corresponding empty vectors were used to transform the *bcs1Δ* yeast strain, using the lithium acetate method [27] after growth in YPAD medium. For all the experiments, except for transformation, cells were grown in a liquid SC medium (0.69% YNB without amino acids and 0.5% ammonium sulfate (Formedium™, Swaffham, UK), adding 1 g/L dropout mix without uracil) in constant shaking at 28 °C or 36 °C or in solid SC medium using 20 g/L agar for solidification (Formedium™, UK). Media were supplemented with various carbon sources (Carlo Erba Reagents, Cornaredo, Italy) as indicated in the results and figures. For growth analyses, the strains were serially diluted, spotted, and grown at 28 °C or 36 °C on SC medium agar plates supplemented with a fermentable carbon source, 2% glucose, or an oxidative carbon source, 2% glycerol.

#### 4.2.2. Oxygen Consumption Measurement and Enzymatic Activity

Mitochondrial respiratory activity in yeast was evaluated by measuring oxygen consumption, using a Clark-type oxygen electrode (Oxygraph System Hansatech Instruments, Pentney, UK) at 30 °C with 1 mL of air-saturated respiration buffer (0.1 M phthalate–KOH pH 5.0, 0.5% glucose) from yeast cell suspensions cultured for 16 h at 36 °C or 18 h at 28 °C in liquid SC medium supplemented with 0.6% glucose until exhaustion. The activity of the respiratory complex III (NADH-cytochrome c oxidoreductase, NCCR) was measured spectrophotometrically on a mitochondrial-enriched fraction as previously described [28,29].

#### 4.2.3. Measurement of Iron Levels

Cells were grown at 36 °C up to the early stationary phase in SC medium supplemented with 1% glucose and 2 mM of ferrous sulfate. The assay was performed as previously described [16] with minor modifications. Briefly, 3 × 10^8^ cells were collected and washed twice with H_2_O, resuspended in 0.5 mL of 3% nitric acid, and incubated overnight at 95 °C. After incubation, samples were centrifuged at 12,000 rpm for 5 min and the supernatant (400 µL) was mixed with 160 µL of 38 mg/mL sodium L-ascorbate (Sigma Aldrich, Merck KGaA, Darmstadt, Germany), 320 µL of 1.7 mg/mL BPS (ACROS ORGANICS, Thermo Fisher Scientific, Waltham, MA, USA), and 126 µL of ammonium acetate (SIGMA) (saturated solution diluted 1:3). Non-specific absorbance was measured at 680 nm and subtracted from the specific absorbance of the iron–BPS complex (535 nm). Iron was quantified by reference to a standard curve using ferrous sulfate.

#### 4.2.4. Protein Quantification and Localization

Proteins were extracted from yeast cells grown for oxygen consumption assay. For protein quantification, protein extraction was performed with the trichloroacetic acid method; to determine the cellular localization (mitochondrial vs. cytosolic) of BCS1L/Bcs1 proteins, mitochondrial and cytosolic protein fractions were obtained as previously reported [30].

Proteins were detected with primary antibodies for BCS1L protein (1:1000; Elabscience Biotechnology Inc., Houston, TX, USA), HA epitope (1:1000; Roche Applied Science, Basel/Kaiseraugst, Switzerland), Por1 (1:10,000; Abcam, Cambridge, UK), and Pgk1 (1:5000; Abcam) followed by fluorescent secondary antibodies (anti-rabbit StarBright™ Blue 700 1:5000, anti-mouse StarBright™ Blue 520 1:5000, anti-rat DyLight 800 1:5000, Modena, Italy). Signals were detected using Chemidoc MP Imaging System and quantified with Image Lab software (Bio_Rad, Hercules, CA, USA).

### 4.3. Patient-Derived Fibroblast Model

#### 4.3.1. Cell Isolation and Growth

Primary fibroblasts were isolated from a skin punch biopsy from the *BCS1L* patient and a control. After mechanical and enzymatic digestion (collagenase V and trypsin) of the biopsies, the isolated cells were seeded in petri dishes, in vitro expanded, and cryo-stored. Only cells at passage 3–10 were employed in the experiments.

For cell growth experiments, cells 2000 were plated on E-Plate 16 (Agilent Technologies, Santa Clara, CA, USA, 05469830001) and cultured in either in DMEM containing 4500 mg/L of glucose or Galactose 10 mM. Real-time cell growth analysis was performed for 72 h by the xCELLigence RTCA instrument (Agilent).

#### 4.3.2. Oxygen Consumption Measurement and Enzymatic Activity in Fibroblast Cells

Oxygen consumption measurement was carried out in fibroblast cells at the Oroboros high-resolution respirometry (Oroboros Instrument, Innsbruck, Austria).

Two million cells were harvested, resuspended in Mir05 medium (Oroboros instrument), and analyzed at the Oroboros instrument (SUIT-007 O2 pce D030 protocol). After the analysis of routine respiration, the cells were permeabilized with digitonin and subsequentially treated with glutamate (leak respiration), ADP and malate (OXPHOS capacity), mitochondrial uncoupler (electron transfer ET capacity), and antimycin A (residual oxygen consumption). The traces of oxygen consumption were analyzed comparing the control and the patient’s cells.

For experiments performed in absence of glucose, cells were incubated in galactose 10 mM for 16 h prior to oxygen consumption analysis.

The ETC activities were assessed spectrophotometrically in the cells.

About 2 million cells were harvested and permeabilized by digitonin treatment. The proteins in the lysates were quantified by Lowry assay (Bio-rad); then, the Citrate Synthase activity was assessed, and both the measures were employed as the control and normalizer.

The ETC I (NADH dehydrogenase), ETC III (decylbenzylquinonol: cytochrome c oxidoreductase), and ETC IV (Cytochrome c Oxidase) activities were monitored as previously described [31] at the Biotek Cytation 5 cell imaging multimode reader (Agilent Technologies, USA). All the reagents employed in the assays were purchased from Sigma-Aldrich (Merck).

#### 4.3.3. RNA Extraction and Quantitative Real-Time PCR

In total, 250,000 cells were seeded on 6-multi-well plates. After 24 h, they were harvested in TriFast (Euroclone, Pero, Milan, Italy) for total RNA extraction following the manufacturer’s instructions. Analysis of concentration, quality, and purity were assessed with the NanoDrop ND-1000 Spectrophotometer (NanoDrop Technologies Inc. Wilmington, DE, USA). For qRT-PCR analysis, 1 μg of total RNA was retrotranscribed using the iScript™ Advanced cDNA Synthesis Kit (Bio-Rad, Hercules, CA, USA, #1725037). Quantitative gene expression analysis was carried out on obtained cDNAs by using TaqMan probes for *BCS1L* gene (Hs01018008_g1) and for *ACTB* (as calibrator and reference Hs99999903_m1) with the Gene Expression Master Mix on the Real-Time CFX Opus platform (Bio-Rad). Experiments were performed at least three times, and each sample is the average of a technical duplicate. The quantification is based on the 2^−ΔΔCt^ method using ACTB as housekeeping gene levels as the normalization reference [32].

#### 4.3.4. Western Blot Analysis

In cell models, total cell extracts were prepared in RIPA Buffer (89900, Thermo Fisher Scientific) supplemented with Halt Protease and Phosphatase Inhibitor Cocktail (TFS). Protein concentration was determined with Bio-Rad Protein Assay Reagent (Bio-Rad, #500-0006). Lysates were resolved by SDS/PAGE. Western blot analysis was performed with the iBind system (Thermo Fisher Scientific) according to the manufacturer’s instructions.

To perform Blue Native gel electrophoresis, 20 μg of digitonin-permeabilized cell lysate were prepared using the NativePAGE Sample prep kit (Thermo Fisher Scientific, BN2008) and loaded in non-denaturating Bis-Tris Gel 3–12% gradient gels (Thermo Fisher Scientific, BN2011BX10) as previously described [33]. Gels were blotted in PVDF membrane for 30′ with the Trans-Blot Turbo Transfer System (Bio-Rad) using the High Molecular Weight protocol followed by standard WB procedures.

For WB analyses, we exploited anti-BCS1L (Invitrogen™, Life Technologies, Thermo Fisher, Waltham, MA, USA, PA5-96745); anti-RISP (Invitrogen, MA5-34745), anti-Cytochrome-b (Proteintech, Tower One, Singapore, 55090-1-AP), and anti-HSP90 (CST, Danvers, Massachusetts, USA, 4877s) antibodies, and the HRP secondary antibody (A90-116P, A120-101P). Images were acquired using ChemiDoc MP Imaging System (Bio-Rad), and intensity of the bands was quantified using FIJI software v2.14.0/1.54f (NIH Image, Bethesda, MD, USA) [34].

#### 4.3.5. Transfection and Plasmids

In total, 2500 HDFs cells were seeded on a 96-well black optical plate (ECPCR0221 Primo, Euroclone, Italy), and after 24 h were transfected. For DNA transfections, 100 ng of DNA was used together with Lipofectamine LTX transfection reagents (Invitrogen), following the manufacturer’s instructions. Plasmids overexpressing BCS1L were obtained cloning the wt and c-38A>G upstream to FLAG-tag sequence in the pcDNA3.1+/C-(K)-DYK Vector generated by GenScript Biotech Corporation (Piscataway, NJ, USA).

#### 4.3.6. Proximity Ligation Assay (PLA)

In total, 2500 HDFs cells were seeded on a 96-well black optical plate (ECPCR0221 Primo, Euroclone, Italy), and after 48 h they were washed with PBS and fixed in 4% paraformaldehyde (J61899.AK, TFS) for 15 min at room temperature, followed by two washes with PBS. Cells were permeabilized with 0.1% Triton X-100. The PLA was performed using the Duolink In Situ Red Starter Kit Mouse/Rabbit (Sigma, #DUO92101) according to the manufacturer’s protocol [35,36]. The following primary antibodies were used: anti-BCS1L (Invitrogen, PA5-96745), diluted 1:100; anti- Complex III (11A51H12, Invitrogen, 43-9400), diluted 1:50; anti-FLAG tag (clone M2, Sigma-Aldrich F3165), diluted 1:50; anti-Cytochrome-b (Proteintech, 55090-1-AP), diluted 1:50. The cells were directly mounted in wells with the DAPI mounting reagent supplied by the kit and visualized at the Biotek Cytation 5 cell imaging multimode reader, Gen5 Image Prime version 3.10 (Agilent Technologies) and the Ziss LSM900 confocal microscope by Zen 2 Software v3.8 (Zeiss, Oberkochen, Germany). For BCS1L- CIII PLA, and FLAG- Cyt-b 100 or 50 cells for each condition/experiment were analyzed.

#### 4.3.7. Immunofluorescence

In total, 50,000 cells were seeded on glass coverslips, and after 24 h they were stained for 30′ with 200 nM Mito Red (53271, Merck) for mitochondria visualization. Then, the cells were fixed in 4% paraformaldehyde (J61899.AK, TFS) for 15 min, washed in PBS, permeabilized with Triton 0.1% for 10 min and blocked in FBS 3% in PBS for 30 min. Antigen recognition was performed by incubating anti-BCS1L primary antibody for 2 h at 37 °C and with fluorescent Alexafluor secondary antibody for 45 min at 37 °C. Nuclei were counterstained with DAPI (Life Technologies, #10236276001) and the coverslip mounted on glass slides.

The slides were visualized at the Ziss LSM900 confocal microscope by Zen 2 Software v3.8 (Zeiss, Oberkochen, Germany) analyzing on ~50 cells for each condition/experiment. Analysis of confocal images was conducted with the ImageJ mitochondria analyzer v2.3.1 [37].

#### 4.3.8. Statistical Analyses and Reproducibility

All the experiments are representative of at least three independent replicates. Statistical tests were performed using GraphPad Prism 8. *p*-values were obtained using two-tailed Student’s unpaired parametric *t*-test and Mann–Whitney. Reported blot and micrographs are representative of three independent experiments.

## Data Availability

Data are contained within the article or Supplementary Materials.

## Supplementary Materials

The following supporting information can be downloaded at https://www.mdpi.com/article/10.3390/ijms26083670/s1.
