# Supplementary material for: Uncovering a Novel Pathogenic Mechanism of BCS1L in Mitochondrial Disorders: Insights from Functional Studies on the c.38A>G Variant
Source: Int J Mol Sci. 2025 Apr 12;26(8):3670. doi: 10.3390/ijms26083670 (PMC12027322; doi:10.3390/ijms26083670)

Supplemental Table S 1. Scores of The Newcastle Paediatric Mitochondrial Disease Scale (NPMDS) 0-24 months relative to the patient homozygous for the *BCS1L* (NM_004328.5):c.38A>G, p.(Asn13Ser), when assessed at 22 months

**NPMDS section**	**Present patient**	**Maximum score 0-24 months**
**I** (Current Function according to caregiver interview during the preceding 2 weeks)	3	15
**II** (System Specific Involvement according to caregiver interview, clinicians's knowledge and clinical notes during the preceding 6 months)	3	27
**III** (Current Clinical Assessment according to the clinician's examination at the time of assessment or as specified in the question)	6	25
**IV** (Quality of life Questionnaire during the past 4 weeks/past 6 months for the finantial cost concerns due to the disease)	6	25
Overall	18	92

The NPMDS scale through its IV sessions encompasses all aspects of mitochondrial disease. Almast every question in the scale has a possible score from 0-3: 0 representing normal, 1 -mild, 2 – moderate and 3 – severe.

Suppl. FIGURE S1

A

```
BCS1L_Hs_       -----------------------------------MPLSD-FILALKDNPYFGAGFGLVG 24
Bcs1_Sce        MSDKPIDIQYDKQATPNLSGVITPPTNETGNDSVREKLSKLVGDAMSNNPYFAAGGGLMI 60
                                                     **. .  *:.:****.** **:

BCS1L_Hs_       VGTALALARKGVQLGLVAFRRHYMITLEVPARDRSYAWLLSWLTRHSTR-TQHLSVETSY 83
Bcs1_Sce        LGTGLAVARSGIIKASRVLYRQMIVDLEIQSKDKSYAWFLTWMAKHPQRVSRHLSVRTNY 120
                :**.**:**.*:  .  .: *: :: **: ::*:****:*:*:::*  * ::****.*.*
```

B

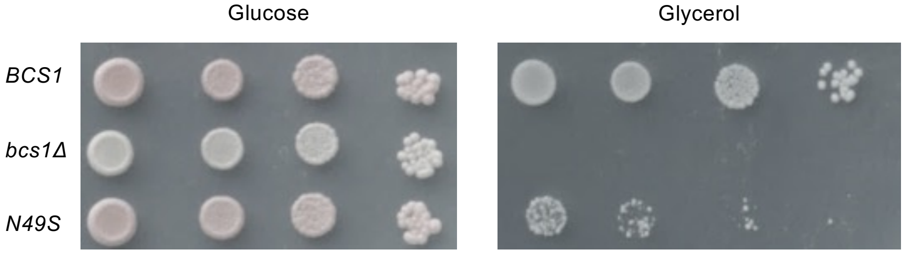

C

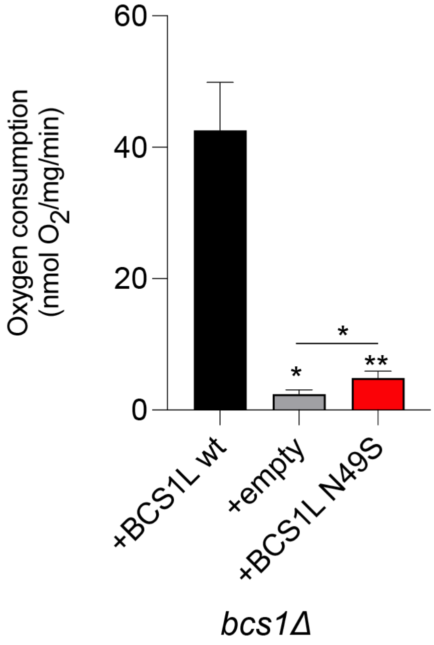

Supplementary FIGURE S2

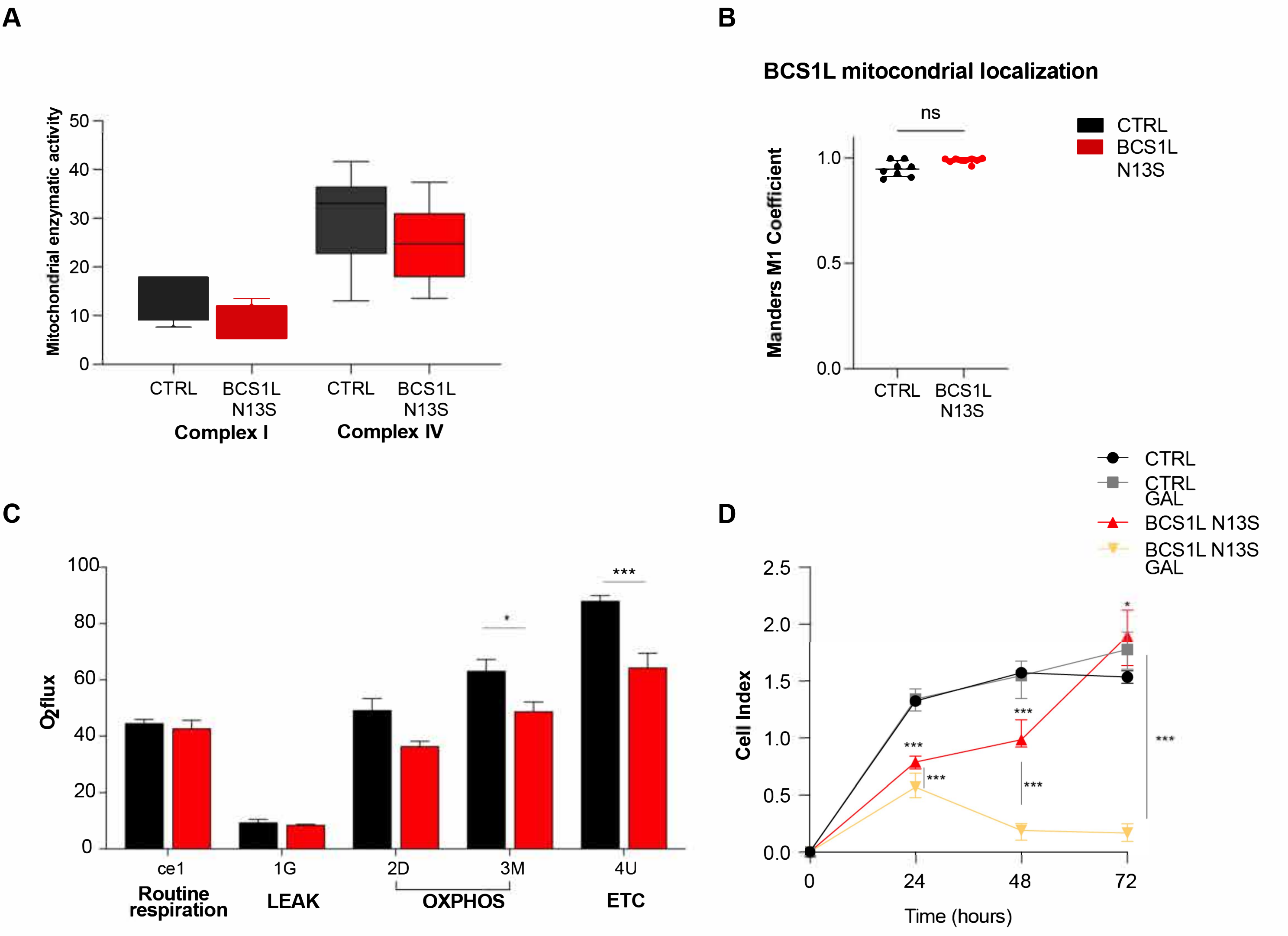

suppl. FIGURE S3

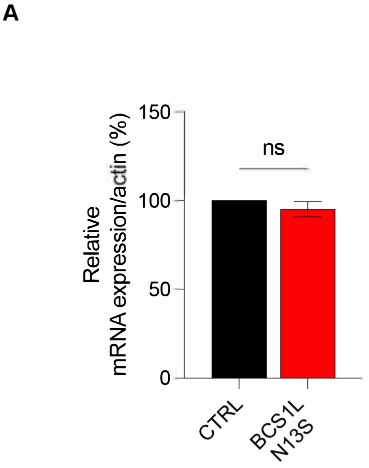

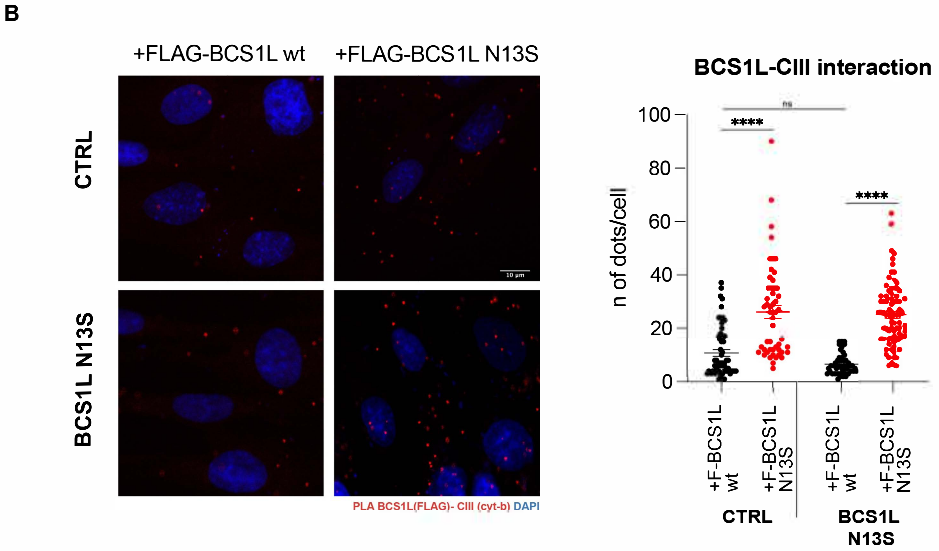

Supplement: Supplementary file 1 [file ijms-26-03670-s001.zip › ijms-3526435-supplementary.pdf]
